# Density Functional Theory Insights into Conduction Mechanisms in Perovskite-Type RCoO_3_ Nanofibers for Future Resistive Random-Access Memory Applications

**DOI:** 10.3390/molecules29246056

**Published:** 2024-12-23

**Authors:** Quanli Hu, Hanqiong Luo, Chao Song, Yin Wang, Bin Yue, Jinghai Liu

**Affiliations:** 1 Inner Mongolia Key Lab of Solid State Chemistry for Battery, Inner Mongolia Engineering Research Center of Lithium-Sulfur Battery Energy Storage, College of Chemistry and Materials Science, Inner Mongolia Minzu University, Tongliao 028000, China; luohanqiong@outlook.com (H.L.); songchao12301@163.com (C.S.); wy19890703@126.com (Y.W.); 2 Department of Chemistry, Tonghua Normal University, Tonghua 134002, China; ybq666666@gmail.com

**Keywords:** cobaltates, density functional theory, perovskite, resistive random-access memory

## Abstract

In the era of artificial intelligence and Internet of Things, data storage has an important impact on the future development direction of data analysis. Resistive random-access memory (RRAM) devices are the research hotspot in the era of artificial intelligence and Internet of Things. Perovskite-type rare-earth metal oxides are common functional materials and considered promising candidates for RRAM devices because their interesting electronic properties depend on the interaction between oxygen ions, transition metals, and rare-earth metals. LaCoO_3_, NdCoO_3_, and SmCoO_3_ are typical rare-earth cobaltates (RCoO_3_). These perovskite materials were fabricated by electrospinning and the calcination method. The aim of this study was to investigate the resistive switching effect in the RCoO_3_ structure. The oxygen vacancies in RCoO_3_ are helpful to form conductive filaments, which dominates the resistance transition mechanism of Pt/RCoO_3_/Pt. The electronic properties of RCoO_3_ were investigated, including the barrier height and the shape of the conductive filaments. This study confirmed the potential application of LaCoO_3_, NdCoO_3_, and SmCoO_3_ in memory storage devices.

## 1. Introduction

In the era of artificial intelligence and Internet of Things, data storage is a research hotspot, which has an important impact on the future development direction of data analysis [1]. The development of data storage is closely related to the development of memory devices. Resistive random-access memory (RRAM) devices are the focus of research in the era of artificial intelligence and Internet of Things [1,2]. RRAM is a promising next-generation memory device with high memory density, fast read and write speed, low power consumption, and compatibility with the existing CMOS technology [3,4,5]. The relentless pursuit of low power consumption, downsizing, economy, and massive storage are the main factors driving RRAM downsizing. RRAM has a wide range of applications, such as consumer electronics, Internet of Things devices, artificial intelligence, memory computing, hardware security, and neuromorphic computing [3,4,5,6]. The RRAM device has a simple sandwich-like structure. When voltage is applied to the electrode, the RRAM device exhibits resistive switching behaviors. The resistance changes reversibly and repeatedly between a high-resistance state (HRS) and a low-resistance state (LRS). The RRAM device can be used in neuromorphic computing architecture to simulate synaptic behavior in human brains, which makes the RRAM device one of the most promising candidates for developing efficient hardware accelerators for artificial intelligence and machine learning tasks, which can significantly improve the speed and efficiency of processing large datasets [2,6]. In addition, the RRAM device can build a three-dimensional cross-storage array, in which storage units can be stacked vertically, effectively enhancing memory density and scalability compared with the traditional planar memory architecture [2]. The reversible resistance switching of RRAM devices between HRS and LRS depends on the formation and breaking of conductive filaments (CFs), which are composed of the cationic motion of electrodes or defects in the resistive switching layer [2,3]. It is reported that the quantization of the conductivity of the conductive filaments can solve the uniformity of the RRAM devices by reducing the charge dispersion effect [7].

Perovskite-type rare-earth metal oxides are common functional materials and considered natural candidates for RRAM devices because their interesting electronic properties depend on the interaction between oxygen ions and transition and rare-earth metals [8,9,10,11]. The rare-earth cobaltates generally present interesting electronic band structures. They can show metallic, semiconducting or insulating properties depending on their composition and crystal structure. The structural flexibility of rare-earth cobaltates allows for the possibility of the doping or substitution of different elements, which can further modify their properties [12,13]. LaCoO_3_, NdCoO_3_, and SmCoO_3_ are typical rare-earth cobaltates. LaCoO_3_ shows an outstanding photoconductive effect and is applied for flexible photodetectors [14]. LaCoO_3_ exhibits remarkable catalytic performance for the oxygen reduction reaction and oxygen evolution reaction on energy storage devices [15,16]. In addition, the competition between high-spin and low-spin states of Co^3+^ endows LaCoO_3_ with an interesting magnetic performance [17]. It is reported that NdCoO_3_ possesses an appropriate bandgap in the visible region and thus exhibits excellent photocatalytic performance on the photodegradation of organic pollutants [18]. NdCoO_3_ and SmCoO_3_ present interesting thermoelectric, electric, and magnetic properties owing to the spin transition of Co^3+^ ions [19,20]. Moreover, the favorable electrical conductivity and energy storage ability of NdCoO_3_ and SmCoO_3_ render them as potential applications for energy storage devices [21]. Oxygen vacancies of perovskite oxides play an important role in improving catalytic oxidation activities [22]. It is also crucial for enhancing the resistive switching performance of RRAM devices [23]. However, the conduction in LaCoO_3_, NdCoO_3_, and SmCoO_3_ for nonvolatile RRAM devices has not been theoretically demonstrated much in the literature. This study aimed to utilize the density functional theory (DFT) to investigate the resistive switching behaviors in RCoO_3_ (R = La, Nd, and Sm) nanofibers. In this study, we proposed that the oxygen vacancies in RCoO_3_ can easily generate conductive filaments, which dominates the resistance transition mechanism of Pt/RCoO_3_/Pt. The electronic properties of RCoO_3_ were investigated, including the barrier height and the shape of the conductive filaments.

## 2. Results and Discussion

Characterization of RCoO_3_

**Figure 1:**
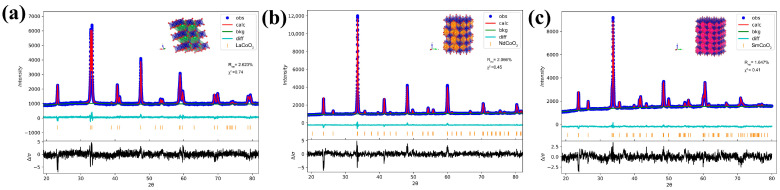
Rietveld-refined XRD patterns of (**a**) LaCoO_3_, (**b**) NdCoO_3_, and (**c**) SmCoO_3_.

XRD Rietveld refinements of X-ray diffraction were carried out to characterize the structures of RCoO_3_. The powder X-ray diffraction patterns of LaCoO_3_, NdCoO_3_, and SmCoO_3_ were plotted, as shown in Figure 1. It is worth noting that the peak intensity is strong, suggesting the high crystallization of the samples. We observed that all characteristic peaks of the obtained LaCoO_3_, NdCoO_3_, and SmCoO_3_ completely matched the standard card of LaCoO_3_ (PDF#97-016-4900), NdCoO_3_ (PDF#97-016-4822), and SmCoO_3_ (PDF#97-029-1093). This indicated the successful fabrication of single-phase RCoO_3_. The lattice parameters are summarized in Table 1. In the Rietveld refined XRD patterns, the solid blue dots represent the experimental data points (obs), the solid red line is the calculated pattern (calc), the bottom green line signifies the background line (bkg), the solid cyan line is the difference between the observed and calculated intensities (diff), and the orange vertical bars are Bragg peak position. 

**Figure 2:**
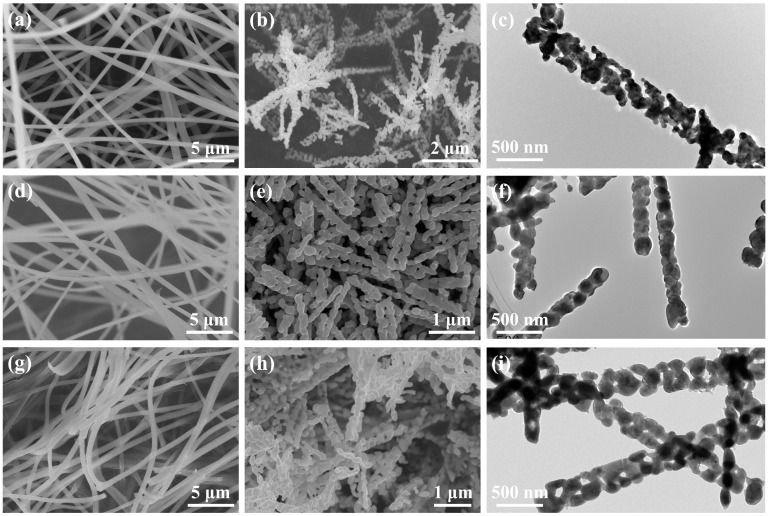
SEM images of (**a**) La(NO_3_)_3_-CoAc/PVP precursor NFs and (**b**) LaCoO_3_ NFs. (**c**) TEM image of LaCoO_3_ NFs. SEM images of (**d**) Nd(NO_3_)_3_-CoAc/PVP precursor NFs and (**e**) NdCoO_3_ NFs. (**f**) TEM image of NdCoO_3_ NFs. SEM images of (**g**) Sm(NO_3_)_3_-CoAc/PVP precursor NFs and (**h**) SmCoO_3_ NFs. (**i**) TEM image of SmCoO_3_ NFs.

Figure 2a,d,g are typical SEM images and show the morphology of the precursors fabricated by the electrospinning method. According to these images, it exhibited a non-oriented fiber network. The precursors have a well-defined nanofibrous structure with a length of tens of micrometers and a diameter of hundreds of nanometers. In addition, these nanofibers are uniform, glossy, and continuous. During the calcination process, the nitrates, acetates, and PVP are thoroughly decomposed, and the nanofibrous morphology of the precursors is destroyed; then, the RCoO_3_ is further produced. Figure 2b,e,h are the SEM images of LaCoO_3_, NdCoO_3_, and SmCoO_3_, uncovering the formation of short nanofibers with average diameters of 370, 190, and 220 nm and an average length of 2–4 μm. To further explore the detailed morphology, TEM analyses were carried out and displayed in Figure 2c,f,i. It is obvious that the surface of LaCoO_3_, NdCoO_3_, and SmCoO_3_ is rough. Moreover, the nanofibers are composed of abundant irregular grains and present a chain-like structure.

**Figure 3:**
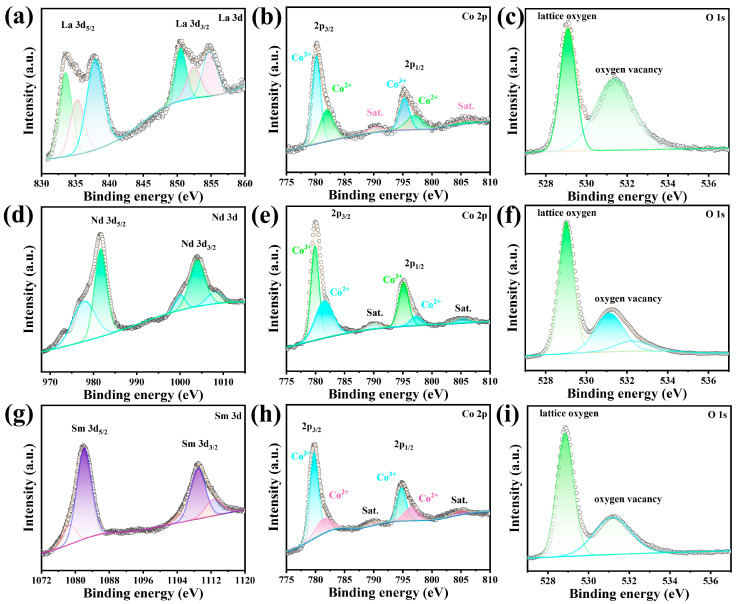
XPS spectrum of (**a**) La 3d, (**b**) Co 2p, (**c**) O 1s, (**d**) Nd 3d, (**e**) Co 2p, (**f**) O 1s, (**g**) Sm 3d, (**h**) Co 2p, and (**i**) O 1s.

The chemical composition and valence states of LaCoO_3_, NdCoO_3_, and SmCoO_3_ were clarified by XPS analyses, and the XPS spectra are displayed in Figure 3. The La 3d spectrum is shown in Figure 3a. The main peaks at binding energies of 833.6 and 850.5 eV indicate the spin orbital splitting peaks of La 3d_5/2_ and La 3d_3/2_, respectively. And the two main peaks with a binding energy difference of 16.9 eV demonstrate the existence of La^3+^ in the LaCoO_3_ [24]. The two doublet peaks can be deconvoluted and fitted into six peaks, which are demonstrated in 833.5, 835.3, 837.6, 850.5, 852.5, and 854.6 eV, respectively. The peaks at 837.6 and 854.6 eV correspond to the La 3d_5/2_ and La 3d_3/2_ satellite peaks, respectively. The former satellite peak originates from spin–orbit interactions, while the latter results from the electron transfer from O 2p to La 4f [25]. The weak peaks at 835.3 and 852.5 eV could be due to the partially reduced La ions [26]. Figure 3d shows the Nd 3d spectrum, which were split into two peaks at 981.5 and 1003.8 eV, corresponding to Nd 3d_5/2_ and Nd 3d_3/2_ with three satellite peaks [27]. As shown in Figure 3g, owing to spin–orbit interactions, the Sm 3d spectrum presents two obvious spin orbits for Sm 3d_5/2_ and Sm 3d_3/2_ with binding energy values of 1081.8 and 1109 eV, respectively, proving the oxidation state of Sm^3+^ [28]. The peaks at 1078.6, 1104.7, and 1113 eV correspond to the Sm 3d_5/2_ and Sm 3d_3/2_ satellite peaks, respectively. Figure 3b,e,h are the Co 2p spectra. The two spin–orbit doublets at binding energies of 780.1 eV and 795 eV are attributed to Co 2p_3/2_ and Co 2p_1/2_; the fitting peaks of these two orbits can be fitted into two peaks, signifying the chemical valence states of Co^2+^ and Co^3+^; moreover, two minor shakeup satellites are at 790 and 805 eV [29]. In Figure 3c,i, the spectra of O 1s can be deconvoluted into two peaks at 529 and 531 eV, respectively, which are the typical peak of lattice oxygen (O^2−^) and oxygen defects such as oxygen vacancy [30]. The O 1s spectrum of NdCoO_3_ can be fitted into three peaks, including the lattice oxygen, oxygen vacancy, and adsorbed oxygen (OH^−^) [29].

Work function

**Figure 4:**
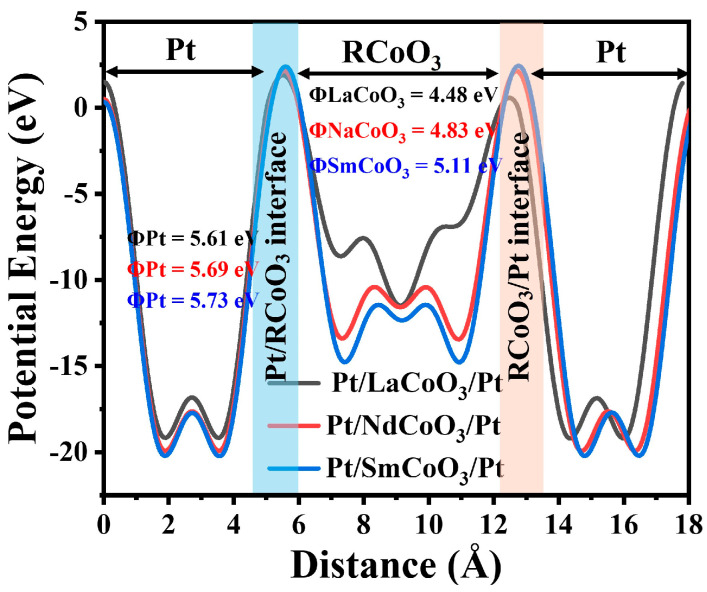
Potential energy line-ups of Pt/LaCoO_3_/Pt, Pt/NdCoO_3_/Pt, and Pt/SmCoO_3_/Pt structures.

The interface between the electrode and the switching layer is the key factor to help clarify the interfacial resistance and conduction mechanisms in RRAM devices [30]. The work function (Φ = E_vac_ − E_F_) can be used to understand interfacial effects. GGA + U calculation was utilized to map up the potential energy lines of the devices with Pt/RCoO_3_/Pt structures, which is presented in Figure 4. It is obvious that the work function value of Pt is much higher than that of RCoO_3_. The work function difference between Pt and RCoO_3_ are Φ_Pt/LaCoO3_ = 1.13 eV, Φ_Pt/NdCoO3_ = 0.86 eV, and Φ_Pt/SmCoO3_ = 0.62 eV, respectively. The charge transfer can modulate the Schottky barrier height by shifting the energy level at the RCoO_3_ and Pt interfaces [31]. The energy barrier between the Pt/RCoO_3_ (RCoO_3_/Pt) interface results in resistive switching behaviors in Pt/RCoO_3_/Pt devices. Under the applied potential, the charges transfer from RCoO_3_ to Pt/RCoO_3_ (RCoO_3_/Pt) interface, leading to space-charge limited conduction [32]. In other words, the charges deplete from RCoO_3_ and accumulate at the interface. The defects like dislocations, vacancies, and interfaces in the switching layer benefit the movement of oxygen across the interface [31]. A large difference in work function can form a strong electric field at the RCoO_3_ and Pt interfaces, which helps to promote efficient charge transfer, thus being conducive to fast resistive switching phenomena by the repeated disrupted and recovered barrier height at RCoO_3_ and Pt interfaces [32]. This is because the difference in the work function causes the electrons to redistribute at the interface and generate an electric field. The enhanced electric field can promote the movement of oxygen ions, because the electric field can apply a force to the positive-charged oxygen vacancies, pushing them along the direction of the electric field, thereby increasing the mobility of oxygen vacancies and further contributing to the conductive filament formation [33]. In addition, the oxidation and reduction of Co ions (Co^3+^ + e^1−^ = Co^2+^) give rise to the charge transfer in the RCoO_3_ switching layer. However, due to the high potential energy of RCoO_3_, charges can be trapped deep inside the RCoO_3_ layer, which may hinder the resistive switching phenomena and lead to a slow resistive switching process [32]. XPS analyses confirmed the existence of oxygen vacancies and Co^2+^/Co^3+^ in the RCoO_3_.

Electronic properties

**Figure 5:**
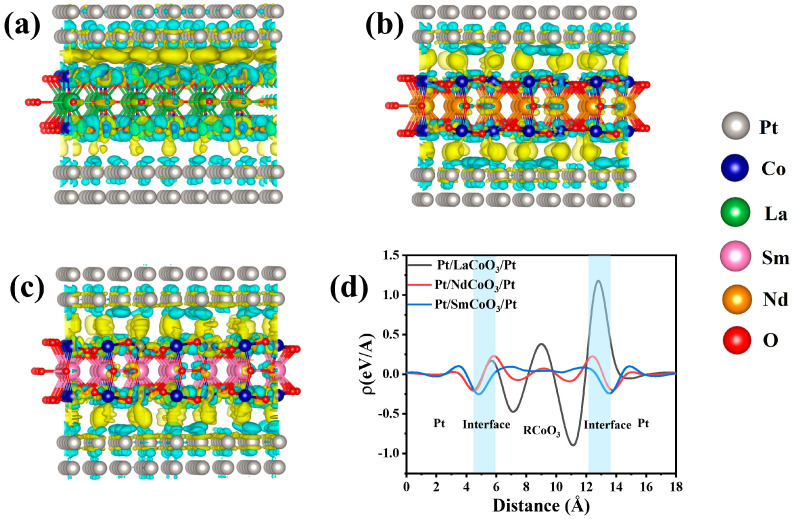
Three-dimensional isosurface charge density plots of (**a**) Pt/LaCoO_3_/Pt, (**b**) Pt/NdCoO_3_/Pt, and (**c**) Pt/SmCoO_3_/Pt. (**d**) Integrated charge density plots of Pt/RCoO_3_/Pt.

To further reveal the electronic properties of RCoO_3_, DFT analyses, including isosurface charge density and density of states (DOS) analysis, were performed at the atomic level to understand the conduction mechanisms of Pt/RCoO_3_/Pt devices. To clarify the charge redistribution phenomena with potential energy lineups, the isosurface charge density and integrated charge density plots along the *c* axis of Pt/RCoO_3_/Pt are displayed in Figure 5. The charge accumulation (yellow color) and depletion (cyan color) of Pt/RCoO_3_/Pt signifies that the charge redistribution occurs at the RCoO_3_ and Pt interfaces. Moreover, the charge transfer resulting from accumulation and depletion suggests the existence of trapping sites in RCoO_3_, thereby highlighting the conduction mechanisms of electron trapping/detrapping in Pt/RCoO_3_/Pt [31]. The irregular (oblate or oblong)-shaped conducting regions around the oxygen atoms across the RCoO_3_ layer uncovers the shape and species of conductive filaments. The “*ρ*” represents the charge redistribution from or to the RCoO_3_ layer. The oxygen atoms in RCoO_3_ play an important role in the charge reduction. As shown in the integrated charge density plots, *ρ* is positive near the RCoO_3_ layer and negative near the Pt layer at the highlighted Pt/RCoO_3_ (RCoO_3_/Pt) interface. It proves the charge transition to Pt layer which has a higher work function. The existence of oxygen vacancies leads to the movement of oxygen atoms toward the vacancies, thus facilitating the conductive filament formation. Unlike Pt/NdCoO_3_/Pt and Pt/SmCoO_3_/Pt devices, the Pt/LaCoO_3_/Pt device presents a positive peak at the RCoO_3_/Pt in the integrated charge density plots, signifying the charge-absorbing action of the Pt electrode, which is beneficial in obtaining a high resistance ratio [34]. Due to the small number of high-value peaks in the integrated charge density plots in the RCoO_3_ layer, the maximum charge is quantized in the irregular-shaped conducting regions around the oxygen atoms. This could be due to the lower potential energy and thermodynamically derived force of the oxygen atoms in RCoO_3_, which causes them to be more inclined to oxidize the conductive filaments [32]. Pt/NdCoO_3_/Pt and Pt/SmCoO_3_/Pt structures exhibit a dispersed charge, implying mediocre resistive switching performance such as a small on/off ratio.

Oxygen vacancies are helpful for charge transfer. Under the applied electric field, the shift in oxygen ions results in the formation of conductive paths along the lattice defects. When there are abundant trap sites in the RCoO_3_ layer, the injected electrons are trapped by these trap sites, and there are no mobile free electrons in the RCoO_3_ layer. Therefore, the resistance is high, and the device is in HRS. When the applied electric field increases, the trap sites are occupied and the charges injected from Pt electrode become the carriers, and the current of the device increases; thus, the device turns into LRS.

Atomic-species-projected density of states

**Figure 6:**
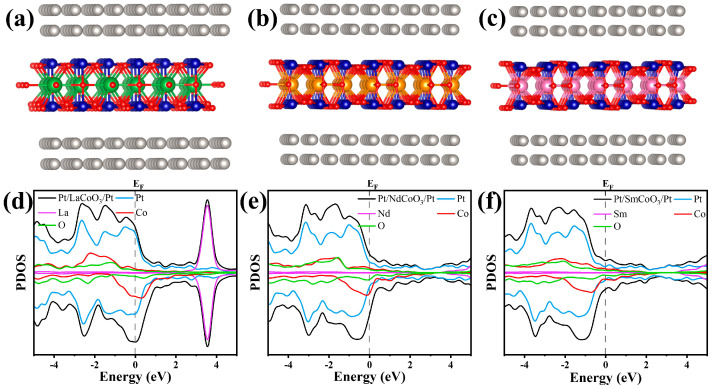
Schematic of a RRAM based on one monolayer of (**a**) LaCoO_3_, (**b**) NdCoO_3_, and (**c**) SmCoO_3_ sandwiched between Pt electrodes. PDOS of (**d**) Pt/LaCoO_3_/Pt, (**e**) Pt/NdCoO_3_/Pt, and (**f**) Pt/SmCoO_3_/Pt.

Atomic-species-projected density of states (PDOS) calculations were used to analyze the electronic properties of Pt/RCoO_3_/Pt structures for the resistive switching memory device application. The conductivity of Pt/RCoO_3_/Pt and each individual atom were clarified by PDOS at and around the Fermi level (E_F_, grey dash line), which is shown in Figure 6. The orbital contribution condition of the Pt/RCoO_3_/Pt structures in the formation of valence band and conduction band regions are summarized in Table 2. According to Figure 6, without regard to Pt atom, the valence band region is mainly occupied by Co 3d and O 2p orbitals, while for the conduction band region, the lower region is dominated by rare-earth orbitals, and the upper region is dominated by Co 3d and O 2p orbitals. The contribution of rare-earth atoms in the valence band region is negligible. In addition, LaCoO_3_ is more conductive than NdCoO_3_ and SmCoO_3_, and the PDOS at the Fermi level decreases from LaCoO_3_ to SmCoO_3_. The PDOS calculations demonstrate that additional states (defect states) exist in the forbidden region. XPS analyses confirmed the presence of oxygen vacancies. These vacancies might lead to the abovementioned additional states, which come from the Co 3d and O 2p orbitals. The multiple states in the bandgap region are formed and split owing to the oxygen vacancies. It indicates that the Pt/RCoO_3_/Pt devices easily switch to a low-resistance state and thus exhibit low power consumption [32]. The interaction between the defect states near the conduction band leads to the accumulation of charge at the interface of RCoO_3_ and Pt. Under the applied electric field, the electrons can be transported through these oxygen vacancy paths, namely the conductive filaments.

Energy barriers

**Figure 7:**
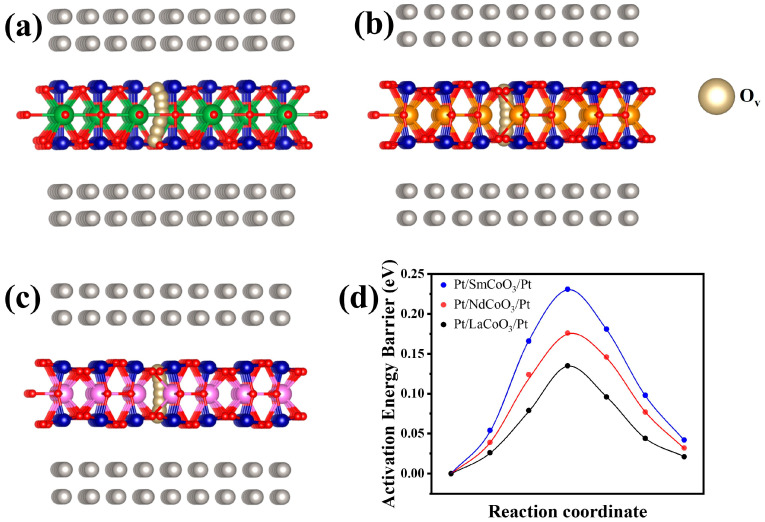
Schematic illustrations of oxygen vacancy paths in (**a**) Pt/LaCoO_3_/Pt, (**b**) Pt/NdCoO_3_/Pt, and (**c**) Pt/SmCoO_3_/Pt. (**d**) Energy barrier of formation of oxygen vacancy path in Pt/RCoO_3_/Pt.

DFT simulation is used to calculate the energy needed to form a conductive filament composed of oxygen vacancies to trigger the low-resistance state. As shown in Figure 7, the energy barriers of the oxygen vacancy diffusion for Pt/LaCoO_3_/Pt, Pt/NdCoO_3_/Pt, and Pt/SmCoO_3_/Pt structures are 0.135, 0.176, and 0.231 eV, respectively. The RCoO_3_ layer contains the oxygen vacancies, and these vacancies tend to connect with others because the energy barrier is very small.

During the SET process, as the device changes from HRS to LRS, conductive filaments composed of oxygen vacancies are formed due to the migration of oxygen ions from RCoO_3_ to Pt. During the RESET process, the device turns into HRS, and conductive filaments are ruptured, owing to the transportation of oxygen ions from Pt to RCoO_3_. The existence of oxygen vacancies in RCoO_3_ facilitates the formation and rupture of conductive filaments. It is worth noting that oxygen vacancies play an important part in the formation of conductive filament and are necessary for the Pt/RCoO_3_/Pt structure to achieve a low-resistance state.

## 3. Experimental and Methods

LaCoO_3_, NdCoO_3_, and SmCoO_3_ nanofibers were synthesized by electrospinning and calcination at 700 °C [35,36,37]. The crystal structure, morphology, microstructure, surface composition, and chemical states of LaCoO_3_, NdCoO_3_, and SmCoO_3_ nanofibers were examined by X-ray diffraction spectroscopy (XRD), scanning electron microscopy (SEM), transmission electron microscopy (TEM), and X-ray photoelectron spectroscopy (XPS), respectively. Moreover, XRD Rietveld refinement analyses were performed using GSAS program.

This research aimed to demonstrate the resistive switching performance in LaCoO_3_, NdCoO_3_, and SmCoO_3_ nanofibers. The density functional theory (DFT) calculations with the VASP code were utilized in this research [38]. The temperature for DFT calculations is 298.15 K. The exchange-correlation was realized by the Perdew–Burke–Ernzerhof (PBE) functional within generalized gradient approximation (GGA) [39], while the projector augmented-wave pseudopotential (PAW) with a kinetic energy cut-off of 500 eV was utilized to depict the expansion of the electronic eigenfunctions [40]. Brillouin-zone integration was sampled by a *Γ*-centered 5 × 5 × 1 Monkhorst-Pack k-point. All atomic positions were completely relaxed until energy and force were reached a tolerance of 1 × 10^−6^ eV and 0.01 eV/Å, respectively. The dispersion-corrected DFT-D method was used to consider the long-range interactions [41]. The minimum energy pathway of the diffuse reaction, along with its corresponding activation barrier, was computed by employing the climbing image nudged elastic band method (CI-NEB). In addition, DFT was not used for structure optimization.

## 4. Conclusions

The electrospun RCoO_3_ NFs have rhombohedral and orthorhombic perovskite structures. XPS analyses confirmed the valence states of R^3+^ and Co^2+^/Co^3+^. It also proved the existence of oxygen vacancies in RCoO_3_ NFs. DFT simulations were utilized to analyze the electric properties of the Pt/RCoO_3_/Pt structure. The energy barrier between the Pt/RCoO_3_ (RCoO_3_/Pt) interface contributes to the resistive switching behaviors in Pt/RCoO_3_/Pt devices. The charge transfer caused by accumulation and depletion reveals the existence of trapping sites in RCoO_3_. The conduction mechanisms in Pt/RCoO_3_/Pt are electron trapping/detrapping. According to the integrated charge density plots, Pt/LaCoO_3_/Pt device could present high resistance ratios. The study of conduction mechanisms of RCoO_3_ provides a strong theoretical basis for its application in memory storage devices. Furthermore, among LaCoO_3_, NdCoO_3_, and SmCoO_3_ nanofibers, Pt/LaCoO_3_/Pt stands out as the most promising candidate for RRAM application.

## Figures and Tables

**Table 1:** The fitting lattice parameters of LaCoO_3_, NdCoO_3_, and SmCoO_3_.

	LaCoO_3_	NdCoO_3_	SmCoO_3_
Crystal structure	rhombohedral	orthorhombic	orthorhombic
Space group	*R* $\bar{3}c$	*Pbnm*	*Pbnm*
*a*	5.44159 Å	5.34849 Å	5.28927 Å
*b*	5.44159 Å	5.33651 Å	5.35376 Å
*c*	13.10618 Å	7.55291 Å	7.50440 Å
*α*	90°	90°	90°
*β*	90°	90°	90°
*γ*	120°	90°	90°
V	336.09 Å^3^	215.57 Å^3^	212.51 Å^3^
*R_wp_* *	2.623%	2.006%	1.647%
*χ* ^2^	0.74	0.45	0.41

* *R_wp_* is the weighted pattern factor.

**Table 2:** Orbital contribution states of Pt/RCoO_3_/Pt structures.

Name of Structure	Orbital Contribution														
	Valence Band Region					Conduction Band Region									
						Lower (3~4.5 eV)					Upper (0~3 eV)				
	La	Nd	Sm	Co	O	La	Nd	Sm	Co	O	La	Nd	Sm	Co	O
Pt/LaCoO_3_/Pt	−	−	−	3d	2p	5d	−	−	−	−	−	−	−	3d	2p
Pt/NdCoO_3_/Pt	−	−	−	3d	2p	−	4f	−	−	−	−	−	−	3d	2p
Pt/SmCoO_3_/Pt	−	−	−	3d	2p	−	−	4f	−	−	−	−	−	3d	2p

## Data Availability

The authors confirm that the data supporting the findings of this study are available within the article. In addition, the datasets used and/or analyzed during the current study are available from the corresponding author upon reasonable request.
